# Regional now- and forecasting for data reported with delay: toward surveillance of COVID-19 infections

**DOI:** 10.1007/s10182-021-00433-5

**Published:** 2022-01-18

**Authors:** Giacomo De Nicola, Marc Schneble, Göran Kauermann, Ursula Berger

**Affiliations:** 1 Department of Statistics, Ludwig-Maximillians-Universität München, Munich, Germany; 2 Institute for Medical Information Processing, Biometry and Epidemiology, Ludwig-Maximillians-Universität München, Munich, Germany

**Keywords:** Nowcasting, Forecasting, COVID-19, Generalized regression models, Delayed reporting, Disease mapping

## Abstract

Governments around the world continue to act to contain and mitigate the spread of COVID-19. The rapidly evolving situation compels officials and executives to continuously adapt policies and social distancing measures depending on the current state of the spread of the disease. In this context, it is crucial for policymakers to have a firm grasp on what the current state of the pandemic is, and to envision how the number of infections is going to evolve over the next days. However, as in many other situations involving compulsory registration of sensitive data, cases are reported with delay to a central register, with this delay deferring an up-to-date view of the state of things. We provide a stable tool for monitoring current infection levels as well as predicting infection numbers in the immediate future at the regional level. We accomplish this through nowcasting of cases that have not yet been reported as well as through predictions of future infections. We apply our model to German data, for which our focus lies in predicting and explain infectious behavior by district.

**Supplementary Information:**

The online version contains supplementary material available at 10.1007/s10182-021-00433-5.

## Introduction

The infectious disease known as COVID-19 hit the planet in tsunami-like fashion. The first cases were identified in December 2019 in the city of Wuhan, China, and by March 2020 infections had already spread over the entire world. Nearly all of the affected countries progressively implemented measures to slow down the spread of the virus, ranging from recommended social distancing to almost complete lockdowns of social and economic activity. These measures eventually proved to be effective, as the number of infections could be slowed down (see e.g., Flaxman et al. 2020 and Roux et al. 2020). This allowed numerous states to relax restrictions, in an attempt to gradually return to normality. At the same time, with the threat posed by the virus still looming, decision makers are forced to strike a balance between epidemiological risk and allowance of socioeconomic activity. In this context, surveillance of the number of new infections became increasingly important, and particularly so on a regional level. Given the local nature of the phenomenon (see e.g., Gatto et al. 2020 and Li et al. 2020), such regional view appears to be of crucial importance. One of the difficulties lies in the fact that exact numbers of infections detected on a particular day are only available with a reporting delay of, in some cases, several days, which occurs along the reporting line from local health authorities to the central registers. The following paper provides a stable tool for monitoring current infection levels, correcting for incompleteness of the data due to reporting delays. This approach is also extended toward predicting new infections for the immediate future at the regional level.

More specifically, the scope of our model is threefold: Firstly, we aim to understand the current epidemiological situation as well as to comprehend the association between detected infections, demographic characteristics and geographical location. Secondly, our goal is to nowcast infections that have already been observed but have not yet been included in the official numbers. New infections are detected through tests and registered by the local health authorities, which in turn will report the numbers to national authorities with an inevitable delay. Since we observe reports of infections for each day, we are able to model this delay, which indeed allows to nowcast infection numbers correcting for infections which have not yet been reported. Note that we are not modeling the incubation period (Qin et al. 2020; McAloon et al. 2020), nor the time passing from the onset of symptoms to detection and registration by the local health authority (Lima et al. 2020), as those are beyond the scope of this paper. We instead focus solely on the delay which occurs along the reporting chain from local to national authorities. Lastly, our aim is also to forecast the epidemiological situation for the immediate future. We here want to stress that our model is not aiming to exactly predict future infection numbers, as that would not be realistic. The goal is rather to give a general idea of what is going to happen in the next days in the different districts, and, perhaps most importantly, help in identifying which districts are going to be the most problematic. This could also help policymakers in making decisions regarding the implementation of safety measures at the regional level. We apply our modeling approach to explain and predict numbers of registered COVID-19 infections for Germany by district, age group and gender. While the regional component is of evident and paramount importance, the age group and gender distinctions are also very relevant, given the powerful interaction of demography and current age-specific mortality for COVID-19 (Dowd et al. 2020).

Our nowcasting approach can also be used to obtain up-to-date measures of the 7-days incidence, both at the local as well as at the national level. This quantity is often used by authorities to assess how hard a specific area is currently hit by the pandemic, and sometimes, as is the case for Germany, it is also employed as a criterion to decide which containment measures are appropriate (Bundesministerium der Justiz 2021). It is especially important to have up-to-date infection numbers when computing such a measure, as it is inherently evolving on a daily basis. At the time of writing, the index is calculated by German officials with reference to the date of report of each infection by the local health authorities. Given that, as already stated, there are significant delays in the reporting of cases from local authorities to national ones, the resulting figures are consistently underestimating the actual incidence, with the error being potentially quite large and problematic. Our nowcasts offer a simple and stable solution to this issue, providing infection numbers that are already corrected for expected delays.

The statistical modeling of infectious diseases is a well developed scientific field. We refer to Held et al. (2017) for a general overview of the different models. Modeling and forecasting COVID-19 infections has been tackled by numerous research groups using different models. Panovska-Griffiths (2020) discusses whether one or multiple models may be useful for COVID-19 data analytics. Stübinger and Schneider (2020) make use of time warping to forecast COVID-19 infections for different countries (see also Cintra et al. 2020), while Dehesh et al. (2020) utilize ARIMA time series models. Ray et al. (2020) combine forecasts from several different models to obtain robust short-term forecasts for deaths related to COVID-19. Fritz et al. (2021) present a multimodal learning approach combining statistical regression and machine learning models for predicting COVID-19 cases in Germany at the local level. Early references dating back to the first stages of the pandemic are Anastassopoulou et al. (2020) and Petropoulos and Makridakis (2020). In this paper we make use of negative binomial regression models implemented in the mgcv package in R (Wood 2017). This allows us to decompose the spatial component in depth, and obtain district-level nowcasts and forecasts for Germany. Our results confirm the dynamic and highly local nature of outbreaks, highlighting the need for continuous regional surveillance on a small area level.

The rest of the paper is structured as follows: Sect. 2 describes the data, while Sect. 3 frames the problem, presents our model and compares the performance of different model specifications over time, motivating our modeling choices. Section 4 exemplifies surveillance and describes how predictions are performed in practice, showing the results for exemplary dates. Finally, Sect. 5 concludes the paper, highlighting the limitations of this study and adding some concluding remarks.

## Data

As previously anticipated, we focus our analyses on German data. To do so, we make use of the COVID-19 dataset published by the Robert-Koch-Institute (RKI) on a daily basis. The RKI is a German federal government agency and scientific institute responsible for health reporting and for disease control and prevention. It maintains the national register for COVID-19, where all identified cases of the disease are reported from the local health authorities to the RKI. In our analysis we make use of daily downloads of the data, which we have at our disposal starting from April 12, 2020 until December 29, 2020.

**Table 1:**
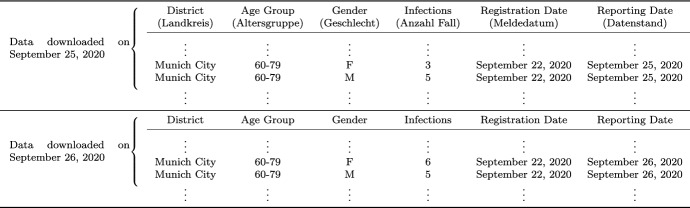
Illustration of the raw data structure, showing downloads of the data from September 25 and September 26, 2020 as an example. To facilitate reproducibility, the original column names used in the RKI datasets are given in brackets below our English notation

**Fig. 1:**
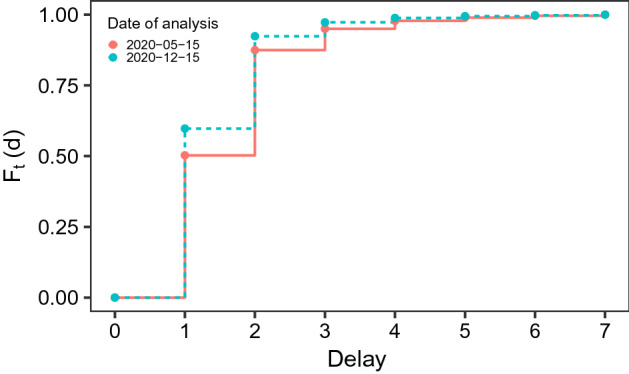
Empirical cumulative distribution function $F_t(d)$ of reporting delays observed during the three weeks preceding May 15 and December 15, 2020

Table 1 shows an exert of the data we are confronted with. Every morning, the database containing all registered COVID-19 infections is updated and released to the public, downloadable from the Robert-Koch-Institute’s repository^1^. The dataset contains, for each of the 412 districts, the cumulated number of confirmed cases of COVID-19 infections stratified by age group (00-04, 05-14, 15-34, 35-59, 60-79 or 80+) and gender, updated to that day. The dataset is also stratified by the date of registration of each case by the local public health authorities (*Gesundheitsämter*). Through the merging of daily downloads of this RKI report, we can construct the full dataset as sketched in Table 1, where the release date is defined in the column “Reporting Date”. This full data format is necessary to trace the reporting delay for each observation. It can sometimes indeed take several days for the data to get from the local health authorities to the nation-wide central one, and we thus define the reporting delay as the number of days between registration date and reporting date. In Fig. 1 we show the empirical cumulative distribution function of the reporting delay observed during the three weeks prior to two exemplary dates close to the extremes of our examined time period. From the plot we can appreciate how the delays were slightly lower in December than in May, possibly due to improvements along the reporting chain. Nonetheless, the delay remains significant across all of our sample. Note that since the RKI reports data every morning, all reported cases will have a delay of at least one day. The delay is especially high during weekends, a fact for which we account in our model. Due to the delayed nature of reporting, the number of registered COVID-19 cases which refer to a specific registration date might change with the reporting date, as exemplified in Table 1. On September 25, 2020, the RKI has reported three registered infections of females in the age group from 60-79 living in the city of Munich, which were registered on September 22, 2020. Due to delayed reporting, this number increased to six in the report of September 26, 2020. The three newly reported cases have therefore been reported with a delay of four days. Note once again that the RKI dataset available for download only contains the information up to the current date, thus making daily downloads of the datasets necessary to determine reporting delay.

For the sake of brevity, we here do not provide general descriptive statistics of the data, since these numbers can be easily obtained from many other sources. Among others, we refer to the RKI webpage^2^, which also includes a dashboard to visualize the data (see also CoronaMaps^3^).

## Surveillance model

### Framing

We start motivating the model by first reformulating the data structure in a way that is suitable for the analysis. Let $N_{t,d}$ denote the newly registered infections at day *t* which are reported with delay *d* and hence included in the database from day $t+d$. The minimum possible delay is one day, and we assume the maximum delay to be equal to $d_{max}$ days. In our analysis we set $d_{max}=7$, which corresponds to a week. In other words, we assume delayed reporting to happen within a week. If we define *T* as the time point of the analysis, the data available at that moment will take the form shown in Table 2.

**Table 2:** Reformulated data structure for a single district, age group and gender, explicitly including delay. Available data are akin to a guillotine blade

t	d			
	1	2	$\cdots$	$d_{max}$
1	$N_{1,1}$	$N_{1,2}$	$\cdots$	$N_{1,d_{max}}$
2	$N_{2,1}$	$N_{2,2}$	$\cdots$	$N_{2,d_{max}}$
$\vdots$	$\vdots$	$\vdots$	$\vdots$	$\vdots$
$T-d_{max}$	$N_{T-d_{max},1}$	$N_{T-d_{max},2}$	$\cdots$	$N_{T-d_{max}, d_{max}}$
$T-d_{max}+1$	$N_{T-d_{max}+1,1}$	$N_{T-d_{max}+1,2}$	$\cdots$	$\text{ NA }$
$\vdots$	$\vdots$	$\vdots$	$\vdots$	$\vdots$
$T-1$	$N_{T-1,1}$	$\text{ NA }$	$\text{ NA }$	$\text{ NA }$
*T*	$\text{ NA }$	$\text{ NA }$	$\text{ NA }$	$\text{ NA }$

**Fig. 2:**
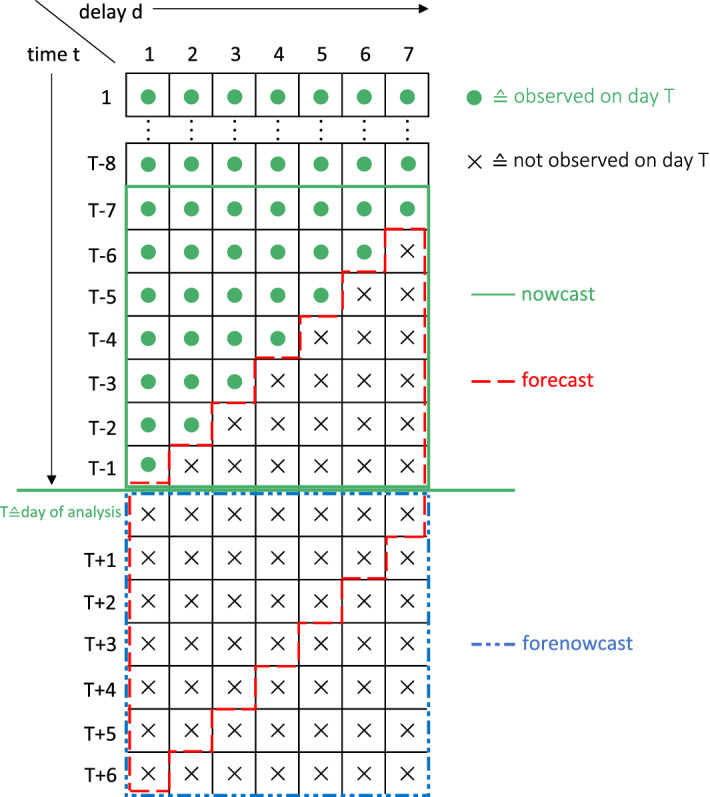
Sketch of the reformulated data structure showing how nowcasting, forecasting and forenowcasting are performed

The bottom right triangle of the data is missing, so that the structure of the available data is akin to that of a guillotine blade. This comparison can be helpful to understand prediction of future values, since predicting by reporting date corresponds to making the blade fall down by one or more days. In other words, one of our goals will be to predict the diagonal edge of the blade, which corresponds to the prediction for cases to be reported on day $T+1$. To better explain our prediction strategy, we give a sketch of this idea in Fig. 2. In the sketch, the green dots represent data that are already observed at time T (the day of analysis), while the crosses represent entries that are not yet observed and that we aim to predict with our model. This is done in three steps, which are described below. To be specific, we pursue *nowcasting*, *forecasting* and the combination of both, which we name *forenowcasting*. Note that forecasting and forenowcasting can be defined, in short, respectively, as "forecasting of reported cases" and "forecasting of registered cases".

*Nowcasting:* Each row of the matrix contains cases registered on a single date and reported with different delays. To obtain the amount of cases registered on that day regardless of the delay with which they were reported we therefore need to take the row sum. If the goal is to obtain predictions by registration date for several days, we then just sum the cases over the corresponding rows. In Fig. 2 we highlight this type of prediction with a green square, which represents a weekly nowcast, that is the number of cases with registration dates over the past week. This comprises numbers that have already been observed as well as the predictions for cases from past days that have not yet been reported.

*Forecasting* If we shift the focus from predicting by registration date to reporting date, that is, if the aim is to predict reported numbers regardless of when the reported infections were actually first discovered, we cannot sum the entries of the matrix row-wise, but we need to do so diagonally. This is because the reported number on day *T* is comprised of the sum of cases registered on day $T-1$ reported with delay 1, cases registered on day $T-2$ reported with delay 2, and so on and so forth, up until cases registered on day $T-d_{max}$ reported with delay $d_{max}$. The red parallelogram in Fig. 2 thus represents the cumulated weekly forecast, that is, the predicted number of infections to be reported over the next seven days. Here all entries are unobserved and will need to be predicted through our model, which will be uncovered in the following section.

*Forenowcasting* We can also combine the two aspects and predict the number of infections that will be registered in the next week, regardless of their reporting date. We call this process “forenowcasting”. While the previously described forecasting (i.e., predicting by reporting date) is useful to get a picture of the numbers that will be reported each day, what really gives a picture of the ongoing situation are infection numbers based on registration date. This weekly prediction corresponds to the blue square in Fig. 2 and in fact is a combination of forecasting and nowcasting. We will demonstrate that the three types of predictions can be carried out with a single model.

### Statistical model

As already stated in Sect. 2, the cumulative numbers of registered COVID-19 infections are, other than by registration date, also stratified by district, age group and gender. To accommodate for this additional information, we extend the notation from above and define with $N_{t,d,r,g}$ the number of newly registered infections on day *t* in region/district *r* and gender and age group *g*, reported by the RKI on day $t+d$ (thus with delay *d*). Row-wise cumulated numbers are defined through

1 $$C_{t,d,r,g} = \sum_{j=1}^d N_{t,j,r,g}$$

which represents the group- and district-specific cumulated number of cases with registration date *t* and delay up to *d*. We define with $\varvec{z}_r$ the geo-coordinates of district/region *r* and generally denote covariates with $\boldsymbol{x}$, where varying subscripts indicate dependence on either gender- and age group *g*, region *r*, time point *t* or delay *d*.

We assume the counts $N_{t,d,r,g}$ to follow a negative binomial distribution with mean $\mu _{t,d,r,g}$ and variance $\mu _{t,d,r,g} + \theta \mu _{t,d,r,g}^2$, where $\theta >0$ and the limit $\theta \rightarrow 0$ leads to a Poisson distribution. More specifically, we set

2 $$\begin{aligned} \mu_{t,d,r,g}& = \exp\{ s_1(t) + s_2(\boldsymbol{z}_r) + {\gamma}_d + \boldsymbol{x}_{t,d} \boldsymbol{\alpha} + \boldsymbol{x} _{g} \boldsymbol{\beta} + {\boldsymbol x}_{t} {\boldsymbol u}_r  \\&\quad + \phi \log(1 + C_{t-1,d,r,g}) + \delta \log(1 + C_{t,d-1,r,g}) + \mbox{offset}_{r,g}\}.\end{aligned} $$

Here $s_1(t)$ is a global smooth time trend, and $s_2(\varvec{z}_r)$ is a smooth spatial effect over the districts of Germany. The parameters $\boldsymbol{\gamma }_d = (\gamma _1, \ldots , \gamma _{d_{max}})$ capture the delay effect for each delay *d*, while the parameters contained in $\boldsymbol{\alpha }$ capture effects related to time and delay, which in our case will be weekday effects. Gender and age effects are included in ${\boldsymbol{\beta }}$, and $\boldsymbol{u}_r$ are unstructured regional effects which will be subsequently specified in more detail. Coefficient $\phi$ captures the time-related autoregressive (AR) component of the process, indicating the effect of cases from the same district and gender- and age group which were registered on the previous day. Coefficient $\delta$ expresses the effect of infections registered on the same day which were reported with delay up to $d-1$, or in other words a delay-related autoregressive component. Finally, the offset is set to the logarithm of the regional population size in the different gender and age groups, enabling us to model the infection rate. Using a population offset is quite standard in disease mapping and in count time series analyses of rare infectious diseases (see e.g., Bauer and Wakefield 2018). The offset defined this way also allows to incorporate the size of the susceptible population in each region, showing that this type of modeling is practicable at different stages of the pandemic. In this case, the population size would need to be replaced by the number of susceptible in region *r*, incorporating the SIR (susceptible-infected-removed) model or other similar ones (see e.g., Allen 1994). This is not particularly relevant at the time point chosen for the analysis, as the number of susceptible corresponds more or less to the population size due to the small (and unknown) size of the immune populations in each district (note that vaccines were not yet available during the analyzed time period).

The previously mentioned spatial effect is comprised of two components: An overall smooth effect $s_2(\boldsymbol{z}_r)$ mirroring the fact that different parts of Germany are differently affected, and a region-specific component accounting for infection rates that are particularly high or low in single districts with respect to the neighbouring situation. To be more specific, $s_2(\cdot )$ is a smooth spatial function of the geo-coordinates $\boldsymbol{z}_r$ for region *r*, while the $\boldsymbol{u}_r$ are unstructured region-specific effects, interacting with the time dependent covariates $\boldsymbol{x}_{t}$. We put a normal prior on $\boldsymbol{u}_r$, i.e., we model $\boldsymbol{u}_r = (u_{r0}, u_{r1})^\top$ as random effects, where ${u}_{r0}$ is a general random intercept capturing the long-term level (from $t = 1,\dots ,T$) of the epidemiological situation in the different districts, while ${u}_{r1}$ is a second random intercept estimated exclusively over the last *k* days, expressing the short-term dynamics (within *k* days prior to $t = T$) of infections. In our analysis we set $k=7$. For $\boldsymbol{u}_r$ we assume the structure

3 $$\boldsymbol{u}_r \overset{iid}{\sim} N(\boldsymbol{0}, \boldsymbol{\Sigma}_u)$$

for $r = 1,\dots ,412$, with the posterior variance matrix $\boldsymbol{\Sigma}_u$ being estimated from the data. The predicted values $\widehat{\boldsymbol{u}}_r$ (i.e., the posterior mode) measure how much and in which direction the infection rate of each district deviates from the global spatial structure, controlling for covariates and age- and gender-specific population sizes.

### Model selection and performance

Model (3.2) includes several components. In this section we aim at assessing whether the inclusion of some of those components is beneficial in terms of predictive performance, and to generally evaluate the overall performance of the final model. Note that we fit the model including only infections with registration dates within 21 days of the day of analysis in the training set. This is because, while on the one hand we would like to use as much data as possible for the fitting, the data-generating process (i.e the spread of the disease) is subject to exogenous changes over time. In other words, we must strike a balance between having a large enough training set and keeping the model as loyal to the current data-generating process as possible. We therefore fit our model using data from a rolling window of 21 days. This choice is motivated more precisely in the supplementary material, where plots comparing the predictive accuracy of the model using different fitting windows are included. The choice of a shorter fitting window also allows to keep other components of the model, such as the smooth spatial effect, constant over time: Such effects are not, in general, time constant, and if we used the whole dataset for the model, we would need to have them interact with the temporal dimension. The rolling window thus also enables the use of a simpler model.

In this section, we are specifically interested in seeing how the unstructured random effects $\boldsymbol{x}_{t} \boldsymbol{u}_r$ and the autoregressive components $\phi \log (1 + C_{t-1,d,r,g})$ and $\delta \log (1 + C_{t,d-1,r,g})$ impact predictive accuracy. To do so, we consider the realized absolute prediction error with regards to nowcasts, forecasts and forenowcasts, cumulated for each district over a period of seven days using different model specifications, to compare performance over time through a weekly rolling window approach. The specifics of how predictions are performed will be described in detail in Sect. 4.

Starting with nowcasting, let therefore $Y_{T,r}^{(n)}$ denote the cumulated number of registered infections in district *r* over $k = 7$ days prior to the day of analysis at time *T*, that is

$$\begin{aligned} Y^{(n)}_{T,r} = \sum _{t=1}^{k}\sum _{g} C_{T-t,d_{max},r,g.} \end{aligned}$$

This corresponds to the sum of all numbers in the green square in Fig. 2. Accordingly, we define with $\widehat{Y}^{(n)}_{T,r}$ the corresponding prediction based on the fitted model as described above. For forecasting, we modify the definition and look at the cumulated number of cases

$$\begin{aligned} Y_{T,r}^{(f)} = \sum _{t = 1}^{k} \sum _{d=1}^{d_{max}} \sum _g N_{T+t-d,d,r,g} \end{aligned}$$

which corresponds to the red parallelogram in Fig. 2. Again, the corresponding predicted value is notated as $\widehat{Y}_{T,r}^{(f)}$. Finally, for forenowcasting we concentrate on the cumulated numbers in the blue square, and set

$$\begin{aligned} Y^{(fn)}_{T,t} = \sum _{t=1}^{k} \sum _g C_{T+t-1,d_{max}, r,g} \end{aligned}$$

with matching prediction $\widehat{Y}^{(fn)}_{T,t}$ based on the fitted model. With the notation just given, we can define the relative district-specific prediction error (standardized per 100,000 inhabitants) simply as

$$\begin{aligned} \text {RPE}_{T,r}^{(\cdot )} = 100\,000 \frac{ Y_{T,r}^{(\cdot )} - \widehat{Y}_{T,r}^{(\cdot )}}{\text {pop}_{r}} \end{aligned}$$

where $\text {pop}_r$ is the population size in district *r*, and the dot refers to nowcasting, forecasting or forenowcasting, respectively. It should be clear that, setting $k = d_{max} = 7$, the numbers defined above are only observable on day $T+7$ for nowcasting and forecasting, and on day $T+14$ for forenowcasting.

To obtain a measure of the overall predictive performance of the model for a certain fitting date *T*, we take the mean of $\text {RPE}_{T,r}^{(\cdot )}$ in absolute value over all districts, which we call Mean Absolute Relative Prediction Error (MARPE):

$$\begin{aligned} \text {MARPE}_{T}^{(\cdot )} = \frac{1}{412}\sum _{r = 1}^{412} {|\text {RPE}_{T,r}^{(\cdot )}|} \end{aligned}$$

To get a sense of the average bias of predictions over time, we also plot the Mean Relative Prediction Error (MRPE), which takes the mean of relative errors without considering them in absolute value:

$$\begin{aligned} \text {MRPE}_{T}^{(\cdot )} = \frac{1}{412}\sum _{r = 1}^{412} {\text {RPE}_{T,r}^{(\cdot )}} \end{aligned}$$

This last measure will be positive if the model tends to underpredict on average over the districts, and negative otherwise.

To evaluate the predictive accuracy of different model specifications, we compute $\text {MARPE}_{T}^{(\cdot )}$ and $\text {MRPE}_{T}^{(\cdot )}$ over time by fitting the model weekly for each of the considered specifications, in a rolling window approach. In particular, we consider te following model variations:

- Full model as in (3.2);
- Model without the time-related autoregressive component, $C_{t-1,d,r,g}$.
- Model without the delay-related autoregressive component, $C_{t,d-1,r,g}$;
- Model without the autoregressive components, $C_{t-1,d,r,g}$ and $C_{t,d-1,r,g}$;
- Model without the short-term district-specific random intercept, $u_{r1}$;
- Model without the unstructured district-specific random effects $\boldsymbol{u}_r$;
- Model without the short-term district-specific random intercept, $u_{r1}$ and the autoregressive components, $C_{t-1,d,r,g}$ and $C_{t,d-1,r,g}$;
- Model without the unstructured district-specific random effects $\boldsymbol{u}_r$ and the autoregressive components, $C_{t-1,d,r,g}$ and $C_{t,d-1,r,g}$;

**Fig. 3:**
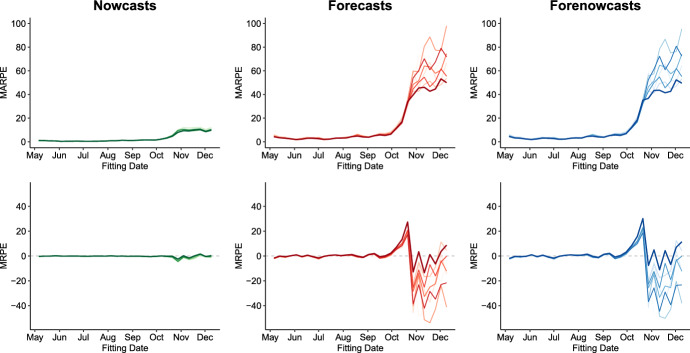
Mean absolute relative prediction error ($\text {MARPE}_{T,r}^{(\cdot )}$, top panel) and Mean Relative Prediction Error ($\text {MRPE}_{T,r}^{(\cdot )}$, bottom panel) for all districts in Germany, calculated over time for different model specifications, respectively, for nowcasts (green), forecasts (red) and forenowcasts (blue). Different color shadings refer to model alternatives. The thicker line indicates the selected model, which corresponds to the full model with the exclusion of the time-related AR component, $C_{t-1,d,r,g}$

Figure 3 plots the MARPE and the MRPE by model fitting date for nowcasts, forecasts and forenowcasts, respectively. The plots already reveal several aspects of the goodness of fit of our model. Looking at the MARPE (top panel), it immediately stands out how the errors for nowcasts are, as expected, much smaller than for forecasts and forenowcasts. Secondly, we can see how prediction errors are remarkably small for the first five months of model fitting. Those months coincide with the late spring and summer months, during which infection numbers were relatively under control in Germany. Our model was thus able to capture most of the variability in the process, resulting in precise predictions not only for nowcasts, but also for forecasts and forenowcasts. Finally, we notice how there is a large increase in MARPE for all fitted models starting from October, which coincides with the beginning of the second wave of COVID-19 in Germany. This is due to the fact that in that period the infection dynamics changed and the numbers got much larger, thus also leading to an increase in prediction errors. The model variant that performed the best during this later period is the full model with the exclusion of the time-related autoregressive component $C_{t-1,d,r,g}$, highlighted with a thicker line. The plots for the MRPE (bottom panel) confirm this fact and help explaining the reasons behind it. For both forecasts and forenowcasts, it is apparent how at the beginning of the second wave all models tend to underpredict, while they overpredict from November onward. The chosen model without the AR component is actually the one which tends to underpredict the most (even though it is not performing worse than the others in terms of MARPE), while it then becomes by far the least overpredicting one in later months. This is beacuse infection numbers grew very fast in October, and models including the autoregressive component were better able to capture the quick increase. In contrast though, after new infections somewhat stabilized, the models including the autoregressive component were still projecting the increase of past months on new ones, causing large overestimation. The chosen model is instead more conservative in its predictions, resulting in better overall predictive performance.

## Applied surveillance

Given that what we propose is a monitoring tool, the results change over time. We here give an exemplary snapshot of the estimates and how predictions can be obtained using Tuesday, September 15, 2020, as date of the analysis. This date was chosen as it lies just before the beginning of second wave of COVID-19 infections in Germany. As an additional remark, note that our analysis is completely reproducible for different dates as well, with code and data openly available and downloadable from our GitHub repository^4^.

### Model-based monitoring

**Fig. 4:**
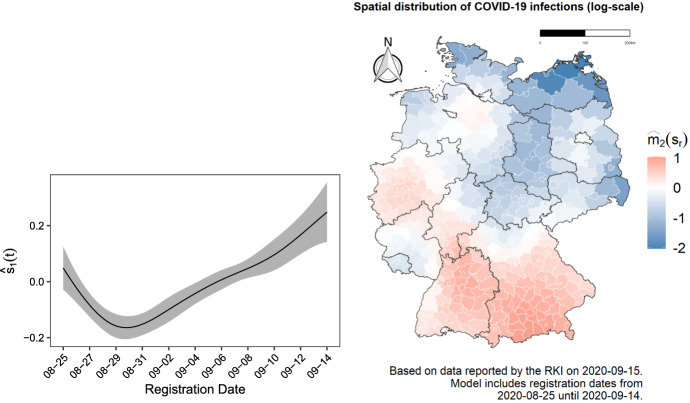
Estimated smooth effects $s_1(t)$ and $s_2(\varvec{z}_r)$, respectively the fitted smooth effect of time and the fitted smooth spatial effect for the prevalence of COVID-19 infections in Germany (measured on the log scale). Both effects are estimated over the 21 days prior to September 15, 2020

In addition to giving proper predictions (nowcasts, forecasts and forenowcasts), which will be shown in the next section, our model also estimates linear coefficients, which are given in table form in the supplementary material, and fits smooth components over time and space, which are visualized in Fig. 4. The left hand side shows the estimated infection rate over time for the three weeks prior to the day of analysis. We notice how the rate of registered infections has been dropping until the end of August, while in the following weeks numbers started rising again, leading to a reversal and a steady increase in the smooth spline. The map on the right hand side depicts the smooth spatial effect estimated as a function of longitude and latitude, on the log scale. From the plot we can see how the regions of Bavaria and Baden-Württemberg in the south of Germany were generally the most affected during the observed period. We also observe that the west was also, on average, more affected than the east.

**Fig. 5:**
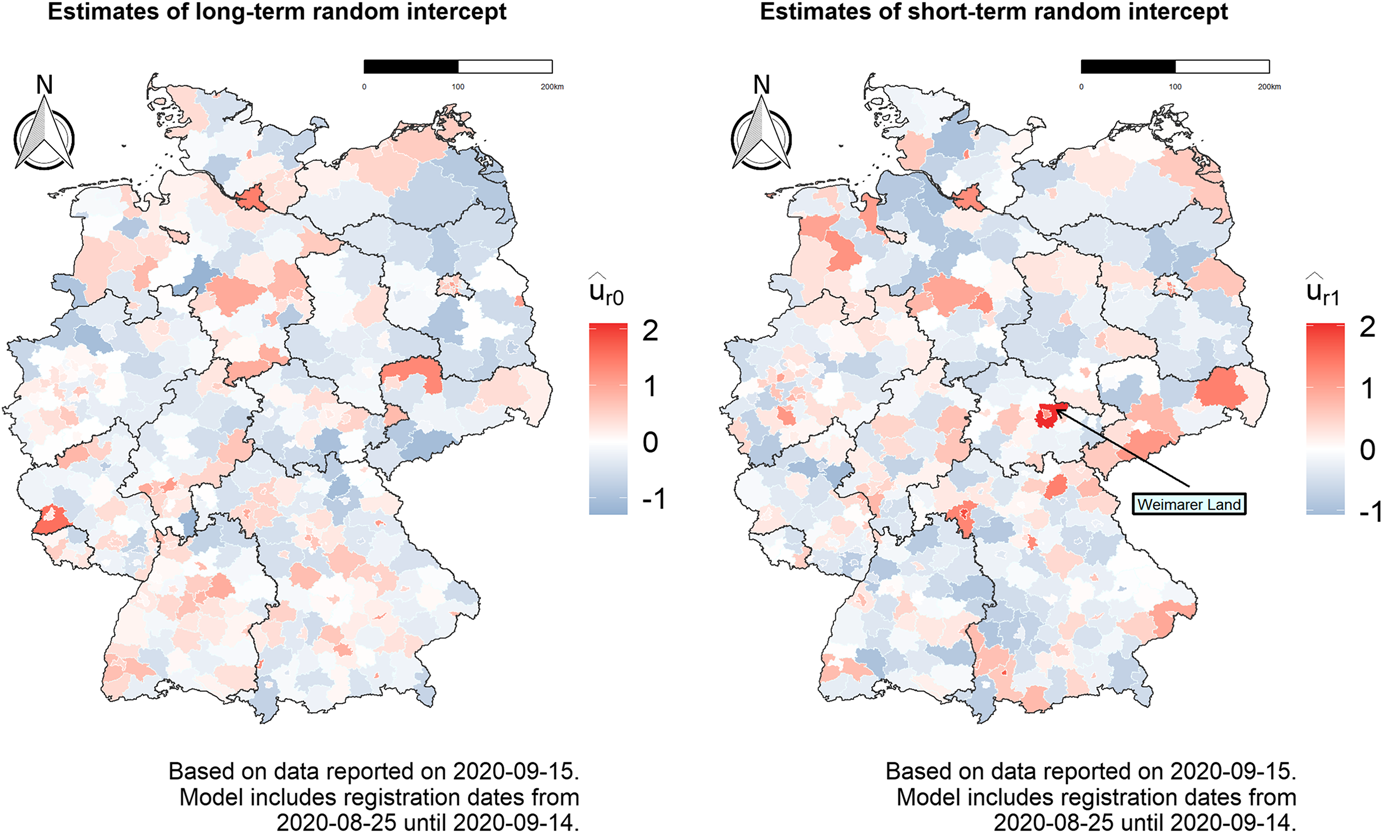
Region specific level (left) and dynamics (right) of COVID-19 infections, controlling for the smooth spatial effect on the right hand side of Fig. 4

The two maps in Fig. 5 show further spatial components of the model, namely the district-specific random intercepts. Those reflect the situation in single districts controlling for the previously shown smooth spatial effect, that is, in comparison to the average of the neighboring areas. More specifically, the map on the left displays the overall district-specific long-term random intercept, depicting the relative infection situation in the 21 days prior to the day of analysis, while the map on the right hand side shows the additional short-term random intercept which enters the linear predictor only over the last 7 days, giving an idea of the more recent infection dynamics. We can thus see that, for example, the district of Weimarer Land in the region of Thuringia has had the most rapidly evolving number of cases in the 7 days prior to the day of analysis controlling for the situation in its surroundings, reflecting the outbreak that happened in the region during the analyzed period. This second map can already be regarded as a first way of monitoring infection dynamics at a local level, even before looking at the predicted numbers: If a district has a very high short term random effect, it probably means that the affected area deserves further consideration.

### Predictions

As previously explained, our model can be used to directly nowcast (correct reports from previous days for delay), forecast (predict the number of cases reported in the next days) and forenowcast (predict the number of infections that will be registered for the next days). The obtained predictions can be used to get a picture of how the pandemic is going to unfold in the short term. In the following, we explain how we obtain those predictions from our model.

*Nowcasting* In our case, nowcasting is equivalent to filling all NA (missing) entries of the matrix in Table 2, turning the trapezoid shape of the data into a full rectangle. This is also equivalent to completing the green square in Fig. 2. Given that we model delay *d* as a stand-alone variable in our generalized additive model, we are able to simply predict the missing cells directly by setting the delay *d* to the necessary value in the data vector used for predictions alongside all other covariates. We can thus nowcast infections for each delay, day, district, gender and age group. If the autoregressive terms $C_{t-1,d,r,g}$ and $C_{t,d-1,r,g}$ are included in the model, the predictions are dependent on them. Those terms are in general not yet known at the day of analysis (except when predicting the first diagonal of the red parallelogram in Fig. 2). We therefore perform the prediction of the black crosses in Fig. 2 iteratively, by utilizing the predictions of the previous diagonal as the autoregressive components. Based on the model, we can also take uncertainty into account by simulating data from a negative binomial distribution with the corresponding mean and variance structure. More precisely, we apply the same strategy as above, but instead of using the mean value we now plug counts simulated from the model into the autoregressive components, and repeat this procedure $n = 1000$ times. This parametric bootstrap approach easily allows us to compute lower and upper bounds of the prediction intervals.

*Forecasting* The model also allows to directly predict cases for future dates. With *T* denoting the time point of data analysis, we can obtain predictions for the number of reported cases on days $T, T+1, \ldots T+k-1$. Let us start with the predictions for cases with reporting date *T*. Referring once again to the guillotine blade structure in Table 2, we proceed as follows: For $d=1$, i.e., at the leftmost point of the blade, we take the fitted mean values as prediction, while keeping the smooth function of time constant, that is, setting $s(t+1) \equiv s(t)$ for the sake of stability. For the remaining $d_{max}-1$ elements of the blade edge we take the mean value by setting $d=d+1$. To get predictions for the numbers of infections reported on days $T+1, \ldots T+k-1$ we can then proceed in an analogous way, using the values just predicted to update the autoregressive components ($C_{t-1,d,r,g}$ and $C_{t,d-1,r,g}$). Figure 2 visualizes the strategy, with cumulated predictions for the number of cases reported on days $T,T+1,\ldots T+6$ being represented by the red parallelogram. Similarly as we did for the nowcasting, we can take uncertainty into account through simulations, sampling from a negative-binomial model with the estimated group-specific mean and variance structure.

*Forenowcasting* Predicting by registration date, i.e., forenowcasting, is equivalent to filling the blue square on the bottom of Fig. 2. This is done by computing forecasts as described in the previous subsection, and then performing nowcasting on the forecasted numbers. We also obtain uncertainty estimates in an analogous way as for forecasts and nowcasts.

### Retrospective surveillance

**Fig. 6:**
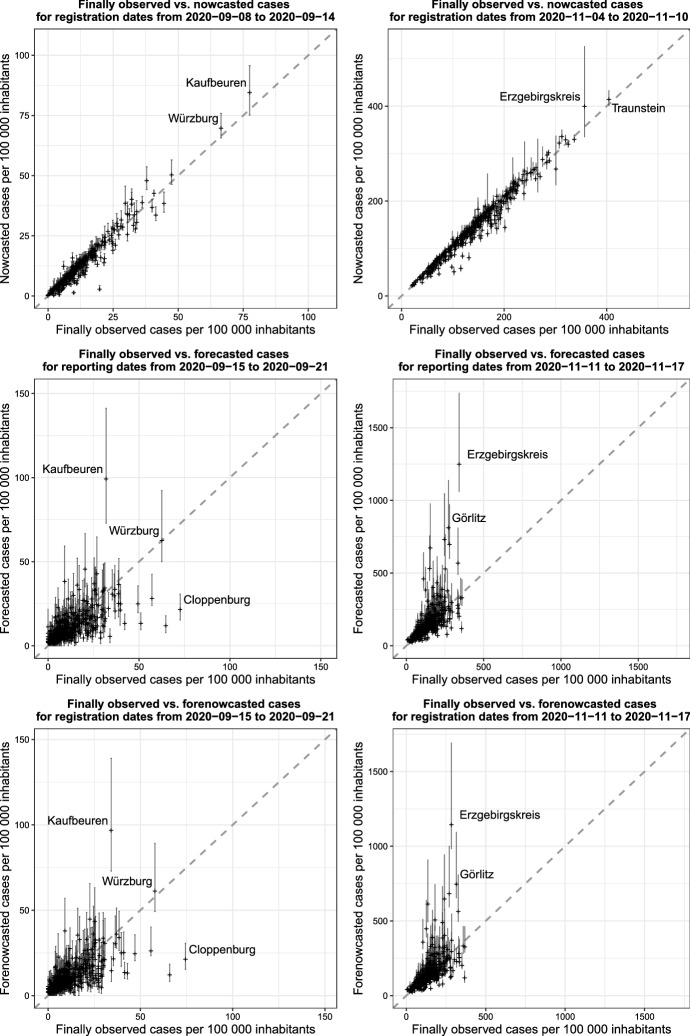
Nowcasts (top), forecasts (middle) and forenowcasts (bottom) of cumulated infections over a week, cumulated by district, plotted against values observed *a posteriori*. The model is fitted with data available on the dates September 15, 2020 (left) and November 11, 2020 (right). Vertical lines represent prediction intervals computed at the 90% level

It is also possible to utilize the proposed model as a surveillance tool retrospectively. After a certain period of time has passed from the day of analysis, we are able to compare predictions with infections observed in the corresponding time span. If the predictions are aggregated on a weekly basis and we keep the maximum delay set as $d_{max}=7$, the waiting time to observe realized infection numbers will be equal to seven days for nowcasts and forecasts and fourteen days for forenowcasts. Figure 6 shows predictions of all three kinds and corresponding infections observed *a posteriori* for two exemplary days of analysis, namely September 15 (left hand panel) and November 11, 2020 (right hand panel).

From the plots we can observe how nowcasts tend to be, in general, quite precise, as already seen from Fig. 3. We can also immediately notice how performance is very different for the two dates, especially for forecasts and forenowcasts: We see that the predictions for September 15 are relatively precise and unbiased, while for November 11 we observe quite a strong tendency toward overprediction.

Focusing first on the forecast and forenowcasts from September (during a “stable” phase of the pandemic), we see that the biggest prediction errors appear for the districts of Kaufbeuren, Bavaria (overprediction) and Cloppenburg, Lower Saxony (underprediction). In the first case, there was an outbreak in a nursing home in the week preceding the forecasted one. This outbreak initially leads to an increase in the infection numbers, but was subsequently contained very quickly, therefore leading the model to overpredict. The underprediction in Cloppenburg, in contrast, was the product of a sudden increase in cases during the forecasted week. More specifically, the higher numbers resulted from cases in schools and the contagion of an almost complete football team in the small city of Löningen. All in all, we can see that in general the prediction errors are not massive, and in line with what we would expect simply due to the inherent randomness of the process.

The situation is different when looking at the predictions for November 11. This is because, while the September date belongs to a period in which the pandemic was relatively stable in Germany, the second one lies at the heart of the second wave. Moreover, the latter date was immediately successive to the sudden increase in new infections in October and to the consequent implementation of social distancing measures (the so-called “lockdown light”) from the beginning of November. However, our model does not include anything regarding exogenous governmental interventions and general changes in population behavior. This means that the predictions are to be interpreted assuming that everything else stays the same as in the three weeks used to fit the model, leading to overprediction for areas in which measures are indeed imposed, and possibly underpredictions after those measures are softened or lifted. As a result, forecasted numbers for November 11 suffer from severe overprediction in many districts. The most extreme example is the district of Erzgebirgskreis, Saxony, which saw a rapid increase in cases between the end of October and the beginning of November, which lead to the district being the one with the highest incidence in the whole country for a short period of time. The infection numbers then stabilized in the following week, leading the model to overpredict. As a side remark, note that the prediction errors for both dates are not majorly spatially correlated. This is in line with our expectations, as both a smooth spatial effect as well as two district specific random effects are included in the model. Maps of the prediction errors by district for both dates analyzed are included in the supplementary material.

While the inability to capture governmental intervention and sudden changes in the population behavior is certainly a limitation of our approach, it can on the other hand also be seen as a feature of the model, which in a sense provides potential future "counterfactual" scenarios in which no action was taken by decision makers. This can thus be used to try to quantify the effect of social distancing policies and interventions, in specific districts as well as at a broader level. This also applies in the case of sudden outbreaks: If a rapid spike in cases in a specific district is observed, and that outbreak was not yet known to health authorities at the time of the analysis, the model will naturally underpredict infection numbers in that district. Severe underpredictions observed *a posteriori* can also be used as an indicator for “true” outbreaks, revealing if they were explainable by past data or not. This “counterfactual” use of our model can thus be seen as an additional feature, which becomes available in retrospect, to measure the effect of NPIs (Non-Pharmaceutical Interventions) and to assess the nature of outbreaks.

## Discussion

We proposed a modeling tool to nowcast and forecast COVID-19 cases reported with delay. This allows to perform surveillance by gender and age group at the regional level, providing an up-to-date and detailed picture of the pandemic, as well as giving insight into the dynamics of the near future. Our model can be used for computing inherently dynamic index measures, such as the 7-days incidence, both at the regional and national level, and it can also aid governments in the implementation of more targeted area- and population-specific containment strategies. However, as previously mentioned, this approach does not come without limitations, which we also want to address.

The number of detected cases greatly depends on local testing strategies and capacities. This implies that comparisons between different states or regions are not straightforward. As our model makes use of reported infections, direct comparisons between outputs should be limited to areas for which it is reasonable to assume that testing has been carried out in a similar manner.

Another important thing to note is that our model only addresses the delay in reporting from local to national health authorities, and not the time that occurs between each test and its (positive) result. This would be useful for our application as it would give an even more up-to date picture of the current situation, but it is not pursued due to a lack of data.

An eminent limitation of our approach is the inability to capture new outbreaks related to specific phenomena that are not yet known to the health authorities. An example of this would be the outbreaks in slaughterhouses which happened during the summer of 2020 in Coesfeld and Gütersloh, North-Rhine-Westphalia. On the other hand, as previously discussed, severe underpredictions observed *a posteriori* can also be used in retrospect as an indicator for outbreaks that are localized and not explainable by past data, while overpredictions can signal and quantify the effectiveness of social distancing measures.

Taking into account the previously mentioned limitations, the model is able to capture a good chunk of the variability that is present. The methodology that we employed is quite general, and, if suitable data are available, can easily be adapted to other countries as well. Moreover, we only employed standard tools for software implementation, and this makes adapting and enriching the model, e.g., with more covariates, relatively straightforward. Our analysis focuses more on now- and forecasting rather than on increasing our understanding of the spread of the disease, and in this context the random effects enable us to capture unobserved heterogeneity fairly well, so the addition of more (time-constant) covariates is not paramount to our goals. Nonetheless it could be fruitful to include more covariates available for specific cases in the model. For the analyzed case of Germany we pursue this in the supplementary material, by adding to the model the German Indexes of Multiple Deprivation, which measure material and social differences at the regional level in Germany (Maier 2017). The results do not differ greatly from what was obtained without this inclusion.

We complete our discussion by emphasizing that the proposed methodology is flexible and applicable to any data constellation in which reporting delay plays a role. In other words, one can easily adopt the proposed model to any guillotine blade-like data structures, i.e., data where $t_i$ denotes the time point of an event and $d_i$ the delay with which the event is reported. Moreover, our approach can not only be applied to correct for the delay between registration of an event and its reporting, but also, for example, to bridge the delay between disease onset and registration of its positive test result. Data in guillotine blade-like form also occur in areas beyond epidemiology, e.g., when cases of unemployment are reported from regional offices to a central state register. The generality of the data structure supports the proposed modeling approach, where corrections for the missing data structure are directly incorporated in the model. In particular, however, the modeling exercise exhibits promising performance for COVID-19 infections, and may therefore be incorporated into a general surveillance tool to assist health authorities and policymakers in their efforts to contain the spread.

## Supplementary Information

Below is the link to the electronic supplementary material.

Supplementary material 1 (pdf 1684 KB)

## Footnotes

1 https://www.arcgis.com/home/item.html?id=f10774f1c63e40168479a1feb6c7ca74

2 https://www.rki.de/covid-19-en

3 https://corona.stat.uni-muenchen.de/maps

4 https://github.com/gdenicola/Now-and-Forecasting-COVID-19-Infections
