# Supplementary material for: Regional now- and forecasting for data reported with delay: toward surveillance of COVID-19 infections
Source: Adv Stat Anal. 2022 Jan 18;106(3):407–26. doi: 10.1007/s10182-021-00433-5 (PMC8764329; doi:10.1007/s10182-021-00433-5)
Supplement: Supplementary file 1 — Supplementary material 1 (pdf 1684 KB) [file 10182_2021_433_MOESM1_ESM.pdf]

Supplementary Material

accompanying the article "Regional now- and forecasting for data reported with delay: Towards surveillance of COVID-19 infections"

Giacomo De Nicola, Marc Schneble, Göran Kauermann, Ursula Berger

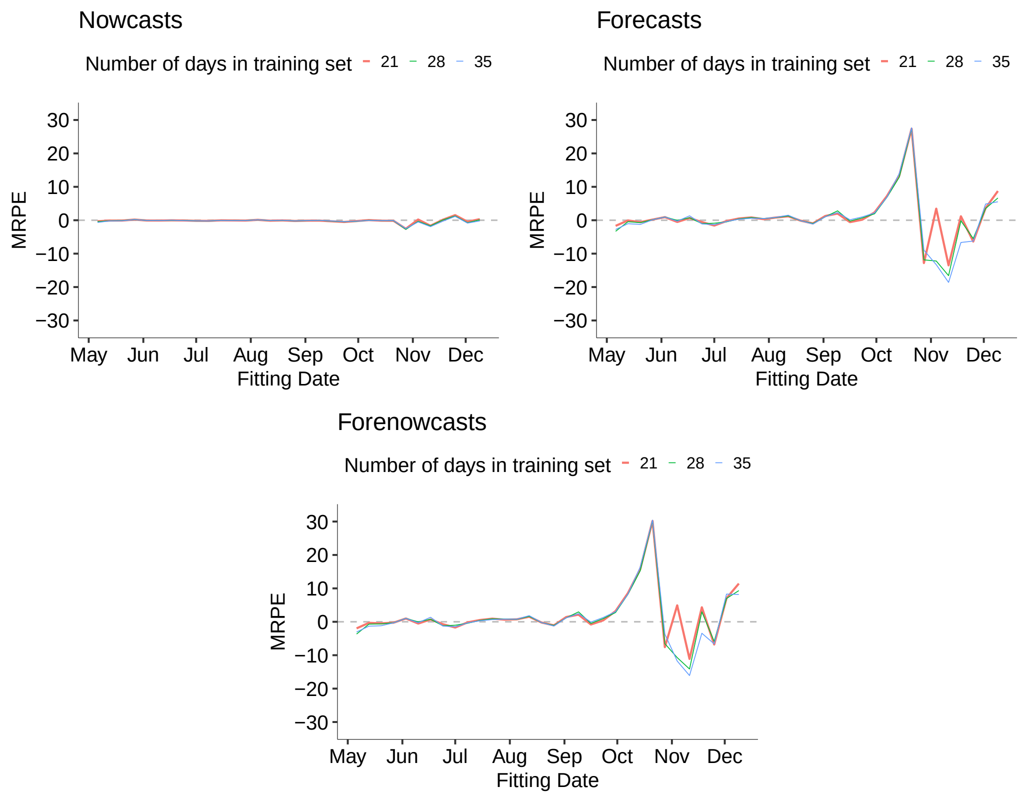

Figure S1: Mean Relative Prediction Error ($\text{MRPE}_{T,r}^{(\cdot)}$) across all districts in Germany calculated over time for different choices of fitting window, respectively for nowcasts, forecasts and forenowcasts. The plots show how the errors are similar until October. Things change from November onwards, as the model fitted with a shorter window (21 days) is able to react quicker to the sudden change in the data-generating process, therefore achieving smaller mean relative errors, particularly for forecasts and forenowcasts. We therefore stick with this relatively short time window.

	Estimate	Std. Error	t value	Pr(> $|t|$)
(Intercept)	-12.241	0.039	-314.73	0.00
Delay 2	-0.037	0.019	-1.93	0.05
Delay 3	-1.514	0.029	-51.36	0.00
Delay 4	-2.432	0.042	-57.24	0.00
Delay 5	-3.209	0.062	-51.84	0.00
Delay 6	-3.872	0.088	-44.23	0.00
Delay 7	-4.589	0.129	-35.66	0.00
Tuesday	0.036	0.039	0.92	0.36
Wednesday	0.184	0.037	4.99	0.00
Thursday	0.148	0.035	4.26	0.00
Friday	0.177	0.033	5.38	0.00
Saturday	-0.242	0.034	-7.12	0.00
Sunday	-0.884	0.038	-23.24	0.00
Age 0-4	-0.701	0.045	-15.75	0.00
Age 5-14	-0.038	0.028	0.92	0.18
Age 15-34	0.740	0.020	37.50	0.00
Age 60-79	-1.040	0.030	-35.17	0.00
Age 80+	-1.206	0.049	-24.38	0.00
Female	-0.083	0.017	-5.03	0.00
log(C.t.d_1)	-0.691	0.025	-27.18	0.00

Table S1: Estimated fixed linear effects for the negative binomial model used for prediction. Parameters and their standard errors are given on the log scale. The reference group for age is 35-59, while the reference category for weekday is Monday. The dispersion parameter is here estimated to be 0.467.

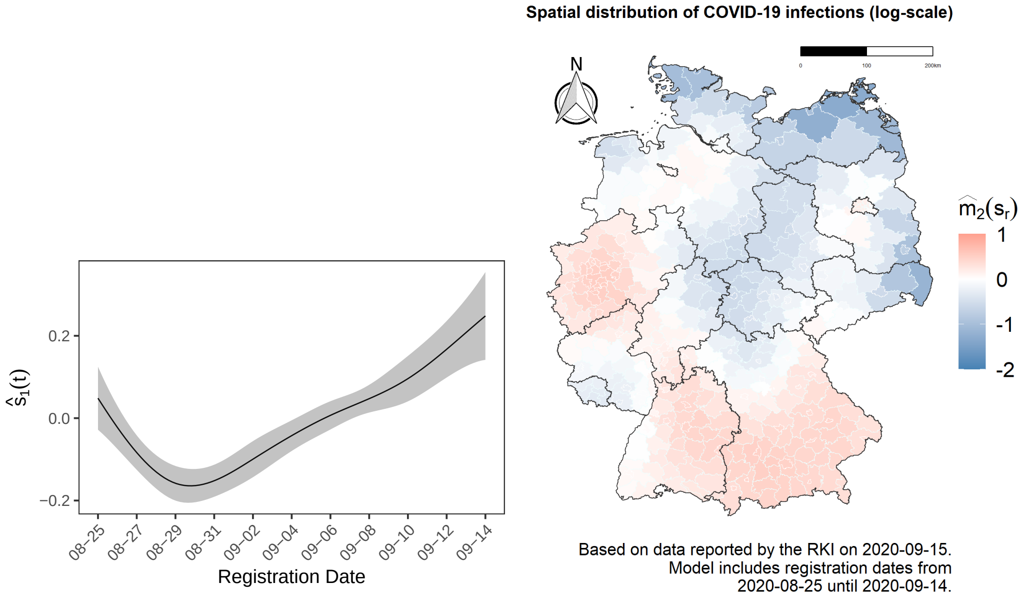

Figure S2: Estimated smooth effects $s_1(t)$ and $s_2(\boldsymbol{z}_r)$, respectively the fitted smooth effect of time and the fitted smooth spatial effect for the prevalence of COVID-19 infections in Germany (measured on the log scale), with the inclusion of the GIMD (German Index of Multiple Deprivation) at NUTS2 level in the model (see the corresponding Eurostat publication for details on NUTS classification levels). the temporal effect has remained pretty much the same, as we would expect. The smooth spatial effect has remained similar in terms of spatial distribution, but decreased in intensity, as the deprivation index captures part of the variability related to the spatial effect. This is presumably due to the fact that, at the date of the analysis, the areas of Germany with the most infections were in southern and western Germany, which are also the areas with lower deprivation indices, while vice versa eastern German states were less affected and have higher deprivation.

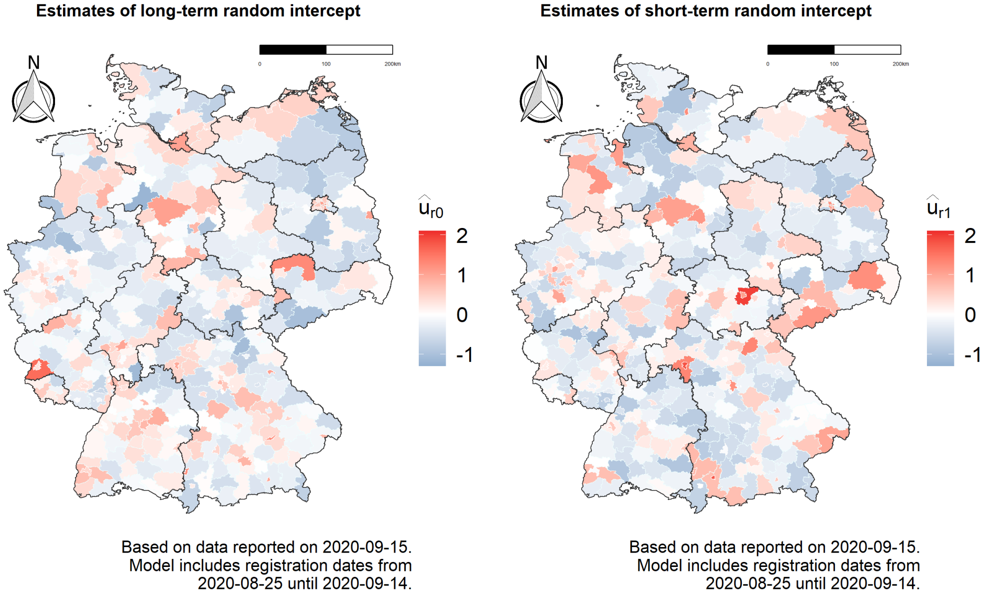

Figure S3: Region specific level (left) and dynamics (right) of COVID-19 infections, controlling for the smooth spatial effect on the right hand side of Figure S2, also obtained with the inclusion of the GIMD in the model. The effects didn't change much with the inclusion of the deprivation index in the model.

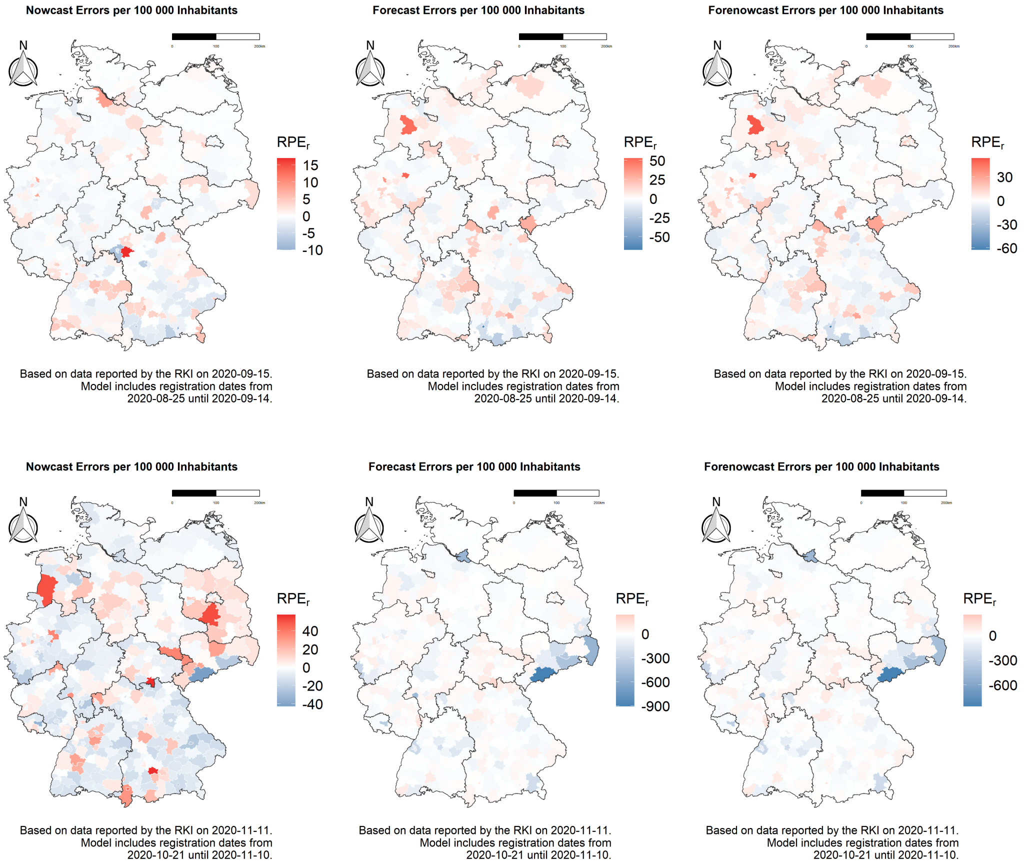

Figure S4: Maps of the relative prediction error by district, $\mathrm{RPE}_r^{(\cdot)}$, for Nowcasts, Forecasts and Forenowcasts, for the dates of September 15, 2020 (top) and November 11, 2020 (bottom). We observe that there is no major spatial correlation between the prediction errors, as both the smooth spatial effect as well as the district specific random effects are included in the model.
